# Intrauterine Exposures and Maternal Health Status during Pregnancy in Relation to Later Child Health: A Review of Pregnancy Cohort Studies in Europe

**DOI:** 10.3390/ijerph18147702

**Published:** 2021-07-20

**Authors:** Chiara Pandolfini, Cristian Ricci, Linda Precious Siziba, Sebastian Huhn, Jon Genuneit, Maurizio Bonati

**Affiliations:** 1 Laboratory for Mother and Child Health, Department of Public Health, Istituto di Ricerche Farmacologiche Mario Negri IRCCS, 20156 Milan, Italy; chiara.pandolfini@marionegri.it (C.P.); maurizio.bonati@marionegri.it (M.B.); 2 Pediatric Epidemiology, Department of Pediatrics, Medical Faculty, Leipzig University, 04103 Leipzig, Germany; linda.siziba@medizin.uni-leipzig.de (L.P.S.); sebastianhu@miltenyi.com (S.H.); jon.genuneit@medizin.uni-leipzig.de (J.G.)

**Keywords:** pregnancy cohort, pediatrics, pediatric epidemiology, prospective studies, public health

## Abstract

We show a description of pregnancy cohorts in the European region. Our investigation identified 66 pregnancy cohorts, mostly hosted in Western Central Europe. Among these 66 cohorts, 24 began recruitment before the year 2000, while six cohorts are still enrolling. The most common topics were lifestyle, environment and nutrition with allergies and neurodevelopment being a minority. We observed a pattern of positive correlations between data collected using medical records, structured interviews, and the collection of biological samples. Objectively assessed data were negatively correlated with self-administered questionnaires. Eight cohorts addressed intrauterine exposure, focusing on environmental pollutants such as endocrine-disrupting chemicals. The effects of these compounds on the developing foetus have been studied greatly, but more research on their effects is still needed. Many cohorts investigated genetics through the collection of biological samples from the mothers and children, to improve knowledge on the mother-to-child transmission of genetic information, antibodies, microbiota, etc. Paediatric epidemiology represents an important field of research since preserving healthy lives from conception onwards is the most efficient way to improve population health. According to our report, it seems that this field of research is well developed in Europe, where numerous high profile studies are currently ongoing.

## 1. Introduction

Birth and pregnancy cohorts are prospective studies aimed at investigating the association between early life exposures and health outcomes later on in life. Of note, pregnancy cohorts recruit women during pregnancy and follow them and their children up, while birth cohorts recruit at birth and usually collect data mostly on the children. Follow-up of participants can continue for years or even for the entire lifespan. The study of the association between early life exposures and health outcomes in later life is often referred to as the study of developmental origins of health and disease (DOHaD). Early life exposures involving many different areas, such as socio-economic, genetic and environmental factors, have been found to influence health later in life [1]. The concept of early life exposure not only refers to newborns and children, but also includes the preconception period, pregnancy and early infancy [2,3].

For example, maternal health in the preconception period, as well as pre-existing maternal diseases and conditions such as diabetes and obesity during pregnancy, have been found to increase the risk of metabolic disorders and cardiovascular disease in later life [4,5]. Moreover, maternal changes during the gestational period, such as excessive gestational weight gain, have been found to be potentially associated with intrauterine growth and/or long-term cardiometabolic health [6]. The correlation of perinatal impacts on neurologic and psychiatric disorders is also being studied [7]. These influences can be explained by the concept of epigenetics, which refers to factors that alter gene expression without changing the primary DNA sequence [7]. The epigenome, described as an interface between the genome and the environment that is central to the generation of phenotypes and their stability throughout the life course, is easily shaped by environmental factors during the perinatal period [8]. The field of DOHaD research has provided ample evidence of the consequences of exposure to risk factors in pregnancy and has helped to implement clinical practices aimed at prevention and to positively influence people’s lifestyles. Much of this research was made possible with prospective pregnancy and child cohorts, and many have been carried out throughout the world, several of which are currently ongoing.

Prospective cohorts are, in fact, the optimal research method for studying this field because exposure precedes outcomes and can be studied prospectively and thoroughly, without obstacles such as recall bias. However, managing cohorts is costly, and long-term follow-up is not always possible. One of the oldest European cohorts is the United Kingdom (UK) Medical Research Council’s 1946 National Survey of Health and Development [9]. In this study, from an initial sample of 16,695 children born in the UK in one week in 1946, a sub-sample of 5362 babies were selected for follow-up, and a wide range of health and functional measures, including cognitive function, have been studied. The UK government’s continuous funding of the cohort has allowed for a life-long follow-up, which still continues. Additional efforts have been made, also at the European Union level, to identify and classify existing cohorts to facilitate collaboration and, possibly, to pool data [10]. One of these initiatives is birthcohorts.net, [https://www.birthcohorts.net, accessed on 10 June 2021] which collects basic information on cohorts and is freely accessible. Single studies have already attempted to combine data from multiple cohorts to obtain more consistent results [11,12,13]. The difficulty in pooling data, however, is in the heterogeneity of data collected, and its format. In this regard, reviews of existing birth and pregnancy cohorts have been carried out to describe the differences and similarities between cohorts. These reviews have focused on different issues, such as types of exposures, specific health conditions, or geographic areas [14,15,16,17,18,19]. One of these reviews recently reported on the current status of European cohorts. The report, however, focused on cohorts that started enrolment at birth, and not in pregnancy, to provide an overview of the current research topics of these birth cohorts and to understand how many addressed the impact of the family context (nurturing care) and the impact of the paediatricians’ care on child health and growth. The final goal was to provide recommendations for future birth cohort studies [19].

Given the centrality of pregnancy cohorts in studying the impact of in-utero environment on epigenetics and offspring health, we decided to amend this report by analysing cohorts that enrolled participants already in pregnancy. We aimed to go into more detail concerning study characteristics, to assess the type and quantity of common data elements collected, and to evaluate what these cohorts addressed in terms of intrauterine exposures and maternal health status during pregnancy and their effects on later child health.

## 2. Materials and Methods

### 2.1. Cohort Identification and Selection

**Figure 1:**
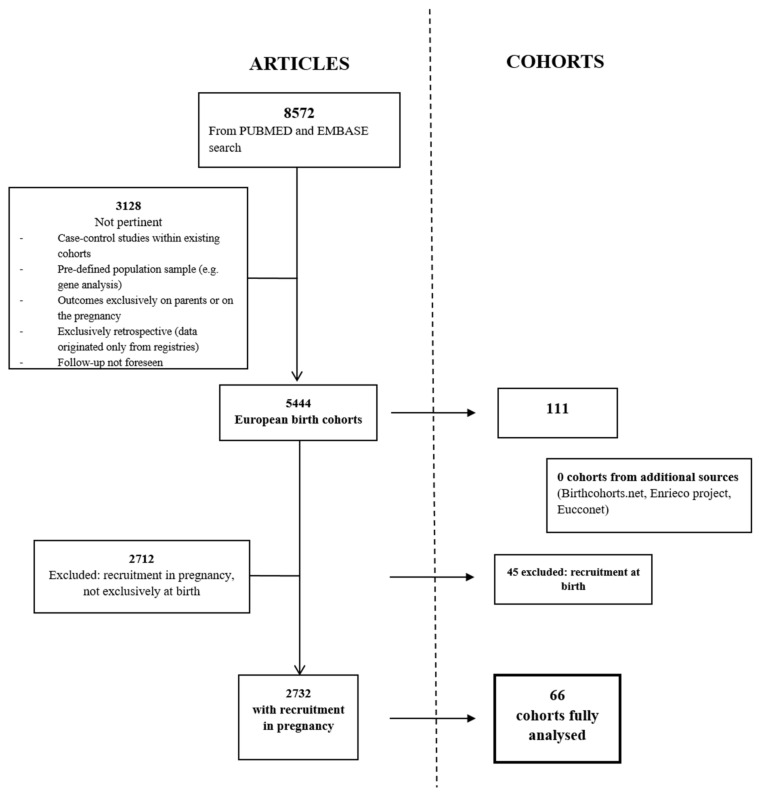
Selection of articles and number of related cohorts. The left panel represent the bibliographic source while on the right panel of the figure the process of cohort selection was reported.

Pregnancy cohorts were selected from a review of the literature published by 20 May 2019 and indexed in the Medline (PubMed) and Embase databases. The details of this literature search have been described elsewhere [19]. Briefly, the two databases were searched for articles referring to birth cohorts based in a European country that collected longitudinal, prospective data on the children. A total of 13,106 records from Medline and 16,087 from Embase were found. After excluding articles based on randomized controlled trials and observational studies focusing on vaccines, genes, or gene expression, as well as duplicate records, 8572 articles remained. The remaining articles were further screened to remove case-control studies designed within existing cohorts, studies that applied gene analysis or other criteria in sample selection, or cohort studies focusing only on the parents or pregnancy outcomes and not addressing the children or that were exclusively retrospective, that collected data from registries, or that did not involve a follow-up. The remaining 5444 articles were reviewed, and the full texts were downloaded when necessary, to obtain the name of the cohort they referred to. In the end, the publications represented 111 cohorts, of which 66 began recruitment already in pregnancy; these were fully analysed. Online cohort inventories such as birthcohorts.net and the chicosproject.eu, and the ENRIECO and EUCCONET networks were also searched for additional cohorts, but none were found (Figure 1). The full text for relevant articles was downloaded for data extraction and, when the publication did not provide sufficient details on the cohort, specific, well-defined steps to search for the necessary information were applied. As such, unless already reported on the official internet webpage of the study, the information was gathered from the protocol paper. If no protocol papers were available then the information was searched for on www.birthcohorts.net. Finally, if still not available, the information was searched on published research papers based on that specific cohort. In this case, since more papers could have been published with the same cohort, we considered the information from the studies with the larger sample size and most recently published as the most authentic.

### 2.2. Data Extraction

Two data frames were used to store the information of the included pregnancy cohorts. One data frame with essential information such as study acronym, the country where the study was conducted, and links to internet resources and more detailed data such as protocol/scientific papers was created. Another data frame stored more detailed information, which was extracted and cross validated by the consensus of four authors (C.R., L.P.S., S.H. and J.G.) using an electronic pilot tested data extraction form based on Microsoft Access^®^ 2016 (Microsoft Company, Redmond, DC, USA). Briefly, the following information was collected for each cohort. General features such as degree of extent on the geographical location (city, region/province, multiple cities and multiple regions/provinces, national or international), a timeline of the data collection (prospective, retrospective or mixed), enrolment start (year), enrolment status (ongoing or closed), the year when enrolment ended, when the enrolment status was closed and eligibility criteria (coded as an open field). Other cohort characteristics collected were the sample size (for both the mothers and children), the follow-up duration, if the follow-up was still ongoing or if it was completed along with an open field reporting the first and secondary aims of the study. The key scientific areas were coded as lifestyle, neuro-developmental, environmental, allergies or others specified in an open field. Finally, the type of data collected from a single cohort was recorded for the children and the mother at pregnancy, at delivery and afterwards. Specifically, the origin of clinical data was classified as clinical examination, structured interview, biological sample, medical files or registries, self-reported, or of another origin (open field). Of note, the data frame was structured with dichotomous variables (yes/no) for each defined level so that a single study could have several different data sources. Moreover, an open field for additional notes was compiled. A further level of detail for data collection was defined according to the use of any other electronic device for non-clinical assessments (GPS location, accelerometery, step-counters, etc.). Information regarding the collection of genetic or biological material along with its origin (blood, cord blood, meconium etc.), previous and current pathologies of the mother or the child and the involvement of twins was collected as well. Finally, other features such as maternal gestational age, funding sources (private, public, hospital consortium, university, foundations or others) and type of operators involved in the data collection (study nurse, paediatrician, gynaecologist) were stored.

### 2.3. Data Synthesis

The number of pregnancy cohorts by country was reported using a map and general characteristics of included cohorts (sample size, key scientific area, type of funding, collection of biological material, etc.) were first grouped merging studies by geographical area. Specifically, cohorts from Sweden, Norway, Finland and Denmark were merged and considered as Northern Europe. England and Eire were kept as a separate insular area while France, Belgium, Netherlands, Germany, Switzerland and Austria were merged as belonging to Western Central Europe. Poland, Slovakia, Croatia, Czech Republic, Ukraine and Lithuania were merged as belonging to Eastern-Central Europe. Finally, Spain, Italy and Greece were merged as belonging to the Mediterranean Area. Time of enrolment and its status (ongoing or closed) were described by a single cohort using a bar chart. The primary aim reported by each cohort, highlighting the investigated association between exposure and outcome, was listed. Finally, to depict the complex association between key scientific areas and the origin of clinical data, a multiple correspondence analysis, a centroid clustering analysis and a network analysis were conducted using the indicator matrix of those features. On one hand, the correspondence and clustering analyses were conducted to depict possible latent factors among the features under investigation and to investigate similarities and recurrent profiles among studies, respectively. On the other hand, the network analysis was conducted to highlight the complex associations among the features under investigation as a whole, taking into account the global picture of correlations. Multiple correspondence and network analyses were conducted using R software version 3.6 using factoMineR and IsingSampler with the Qgraph packages, respectively. Cluster analysis was conducted using PROC CLUSTER and PROC TREE of SAS software version 9.4 (SAS Institute. Inc, Cary, NC, USA).

## 3. Results

### 3.1. Characteristics of Included Cohorts

**Figure 2:**
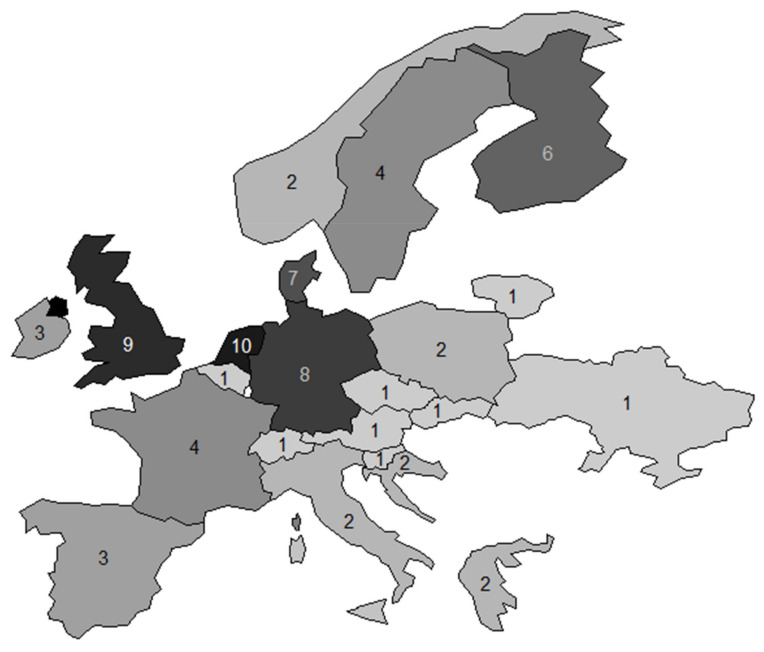
Distribution of pregnancy cohorts by country. Each country is represented using a grey-scale according to the number of pregnancy cohorts. The number of pregnancy cohort is also reported.

**Figure 3:**
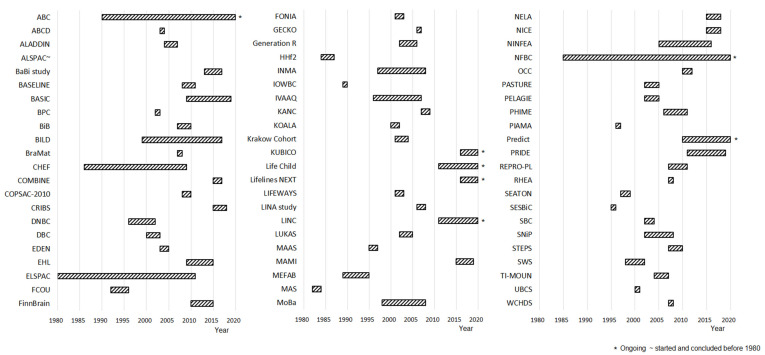
Enrolment starting date and status of the pregnancy cohorts. Pregnancy cohorts were reported by their acronyms as reported in Table 1. Asterisks represent ongoing recruitments.

Among the 66 pregnancy cohorts included, 19 cohorts were conducted in Northern Europe, 12 cohorts were conducted in the U.K. and Eire, 25 cohorts were conducted in Western Central Europe, eight cohorts were conducted in Eastern Central Europe and seven cohorts were conducted in the Mediterranean Area. Netherlands, UK and Germany were the three countries with the largest number of cohorts with ten, nine and eight cohorts each, respectively (Figure 2). An evaluation of the type of cohorts using extent on the territory shows that the majority of cohorts (70%) were conducted at the city or regional level while the smallest part of the included cohorts covered a larger territory. The median sample size of the cohorts was of 1707 mother-child pairs (10th to 90th range = 400 to 11,871). Among the 66 included cohorts, 24 began recruitment before the year 2000, while six cohorts are still enrolling. One included cohort started and finished the enrolment before the year 1980 (Figure 3). The median follow up duration for all cohorts was 30 months (10th to 90th range = 8.4 to 214.8). Among the included cohorts, 11 planned to have a lifelong follow-up. The majority of funding was from the public sector (54%) with a minor contribution from private funding sources (13%). The most common key scientific areas were lifestyle, environment and nutrition representing about 24% of the topic investigated in the included cohorts, with allergy (17%) and neurodevelopment (11%) representing less commonly studied scientific areas of interest. Almost all included cohorts investigated more than one scientific area. Table 1 lists the primary aims reported by the 66 pregnancy cohort studies. It was difficult to identify the cohorts that addressed intrauterine exposure and maternal health because the primary aims were often reported in broad terms. In all, 8 cohorts (BraMat, CHEF, FinnBrain, INMA, Krakow Cohort, MEFAB, PELAGIE, PHIME) specifically mentioned prenatal exposure among their aims, and 1 mentioned maternal health (BASELINE). Some of the 8 cohorts also specified which exposures they aimed to assess, including PFAS, methylmercury and organochlorines, and endocrine-disrupting chemicals in general. Interestingly, 3 cohorts (Lifelines NEXT, which began in 2016; PRIDE Study, 2011; SWS, 1998) specifically mention the study of the pre-conceptional period and its effects on child health in their aims, which is a notion that was over time embraced by the DOHaD concept. The SWS, which began over 20 years ago, is run by the UK MRC’s Lifecourse Epidemiology Centre, an internationally recognised centre for the study of early life influences on later health. Self-reported data were the most common type of data collected on mothers, both in pregnancy and after birth, available in 56% of the studies. With regards to data collected from children, biological samples are collected at birth and during the follow-up by 66.7% and 68.2% of cohorts, respectively. Children’s clinical data were collected at birth by 30% of the cohorts while 57.8% of the cohorts collected children’s clinical data during the follow-up. For mothers, clinical data during pregnancy were collected by 34.8% of the cohorts. Notably, structured interviews during pregnancy were conducted only by 20% of the cohorts. Data from any type of already existing sources such as clinical records or registries were collected by 21.2% of the cohorts. No specific differences emerged when looking at cohorts’ characteristics by geographical area. However, there was a certain degree of heterogeneity with a median sample size ranging between a minimum of 1150 to a maximum of 1800 for Western and Eastern Central Europe, respectively. An evaluation of larger cohorts showed that only two cohorts in Eastern Central Europe and the Mediterranean Area had a sample size above 3000 child-mother pairs. Western Central Europe and UK and Eire (from now on referred to as insular Europe) had five cohorts each with a sample size above 3000 while Northern Europe had six cohorts with a sample size above 3000 child-mother pairs. There was some heterogeneity regarding the cohort start dates, with only 46% of cohorts conducted in Insular Europe started after 2000. Yet, the majority of the cohorts conducted in Western Central (70%), Northern (63%) and Eastern Central Europe (57%) started after 2000. Notably, the majority of the cohorts conducted in the Mediterranean Area started after 2000 (85%).

#### 3.1.1. Collection of the Genetic Information

A total of 26 cohorts collected biological material for genetic analyses of any kind while the other 10 cohorts collected biological material not necessarily for genetic analyses. The most commonly collected biological material was blood (70%), while urine was collected by 39% of the cohorts. Biological material from the umbilical cord and saliva were collected by 29% of these cohorts while breast milk was collected by 27% of the cohorts. Finally, hair and meconium or faeces were less often collected by 14% and 10% of the cohorts. Notably, those cohorts appear as based on large samples with a median sample size of 2854 individuals. Among the 26 cohorts aiming at evaluating genetic material, the majority was established after 2000 (15 cohorts, 58%). In addition, the majority of cohorts assessing genetic material were located in Western Central Europe (12 cohorts, 46%). Among cohorts evaluating genetic material, five were established in insular Europe (19%), four in Northern Europe (15%), While Eastern-Central Europe and the Mediterranean Area contributed with two cohorts each (8%).

#### 3.1.2. Correspondence Analysis Results and Multivariate Clustering of Cohorts

**Figure 4:**
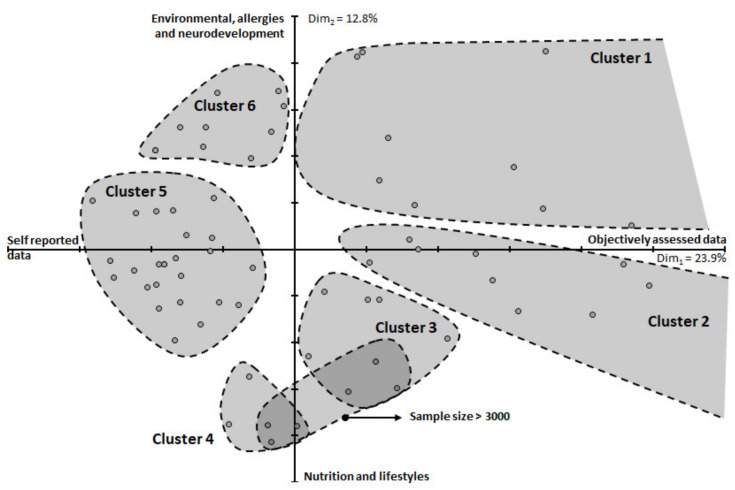
Clustering of the pregnancy cohort according to multiple correspondence analysis applied to research focuses. Grey areas represent results of hierarchical clusters and identify groups of homogeneous studies.

The multiple correspondence analysis applied to key scientific areas and type of data collected resulted in two distinct latent factors; a main and a secondary one related to 23.9% and 12.8% of variance explained, respectively. The main latent factor was related to the dichotomous self-reported vs. objectively assessed measures, with positive loadings related to clinical examination, structured interviews and collection of biological samples. On the contrary, self-administered interviews and data collection from registries were related to negative loadings on this main latent factor. The secondary latent factor instead appeared to be more related to key scientific areas disentangling nutrition and lifestyle from the environment along with allergies and neurodevelopment. The results of cluster analysis and scatter distribution of cohorts over these two latent factors showed at least six clusters of cohorts (Figure 4). The first cluster of nine cohorts (BaBi, COMBINE, TI-MOUN, SNiP, UBCS, Pelagie, LifeChild, MEFAB and IOWBC) sharing a number of different features such as the prevalence of objectively assessed data and being focused on the environment along with neurodevelopment and allergy. Those cohorts are characterized by a heterogeneous sample size ranging between 200 and above 5000. The second cluster of 9 cohorts was derived (FCOU, MAS, LINC, RHEA, PRIDE, BiB, Gen R, ALSPAC and EHL); this cluster had intermediate characteristics by means of scientific key areas but is very focused on objectively assessed data. The sample size of the studies in that cluster ranges from 300 to more than 10,000 children with at least 7 cohorts with more than 1000 participants. A third (COPSAC-2010, ELSPAC, MAAS, PIAMA, Predict Study, SEATON, SWS, WCHDS) and the fourth cluster of cohorts (ABC, CRIBS, DNBC, STEPS, IVAAQ) emerged from the analysis. These clusters comprised eight and five studies, respectively, and generally share the key scientific areas of nutrition and lifestyle. Cluster three and cluster four also share the common features of varying sample sizes ranging between 700 to more than 10,000 individuals. Interestingly, a gradient of sample size was observed running parallel to the second latent factor, in which studies with larger sample sizes were on the lower portion of both clusters. A fifth large cluster of 25 cohorts (ABCD, ALADDIN, BASELINE, BASIC, BPC, BILD, BraMat, EDEN, GECKO, HHf2, INMA, KANC, KOALA, KUBICO, L NEXT, LIFEWAYS, LINA study, LUKAS, MAMI, MoBa, NICE, NINFEA, OCC, PASTURE, SBC) spread on the left side of the bi-plot through the second latent factor. This group of studies had a heterogeneous sample size with the number of mother-child pairs ranging from 200 to more than 10,000. This cluster was mostly associated with the use of self-reported data along with clinical registries while it is quite heterogeneous in terms of the scientific key area. Lastly, a sixth cluster in the upper left part of the bi-plot comprised 10 cohorts with a sample size ranging between 100 to more than 10,000 (CHEF, DBC, FinnBrain, FONIA, Krak C, NELA, PHIME, REPRO_PL, SESBiC, NFBC).

#### 3.1.3. Network Analysis of Key Scientific Areas and Types of Data Collected

**Figure 5:**
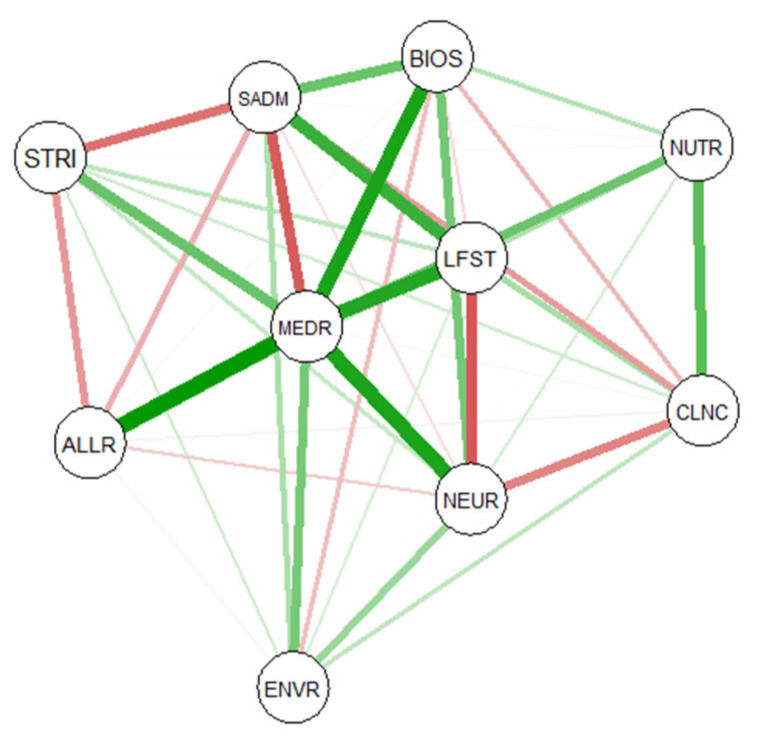
Results from a network analysis of key scientific areas and type of data collected. The size of the line represents the strength of the correlation between two factors. Green and red lines represent positive and negative correlations, respectively. STRI = Structured Interview; SADM = Self-administered data; BIOS = Biological samples; NUTR = Nutrition; LFST = Lifestyle; MEDR = Medical records; ALLR = Allergy; NEUR = Neurodevelopment; CLNC = Clinical assessment; ENVR= Environmental.

Results from the network analysis of key scientific areas and types of data collected showed a pattern of positive correlations between data collected using medical records, structured interviews and the collection of biological samples. Moreover, these two types of objectively assessed data were negatively correlated with self-administered questionnaires. According to our evaluation, the pattern of objectively assessed data was also positively correlated with cohorts investigating environment and neurodevelopment while the association with nutrition seemed weaker, although positive. Network analysis also highlighted the contrast between neurodevelopment/environment and lifestyle/nutrition studies, confirming results from correspondence and cluster analyses (Figure 5).

## 4. Discussion

In the present study, we provide an overview of 66 pregnancy cohort studies in Europe. These studies are considered medium to large cohorts with a median sample size above 1000. Moreover, most of the pregnancy cohorts conducted in Europe seem to have adequate follow-up duration, with a median follow-up duration of more than two years and a substantial part of studies that plan to have a lifelong follow-up. The majority of included cohorts were established after the year 2000 with a considerable number of studies that are currently active. However, the limited number of international cohorts highlights a possible lack of international collaboration. Nevertheless, a large number of high-quality birth cohorts were established in Europe during the last 20 years and differences by geographical areas were generally negligible. This field of study seems to be scientifically sound and potentially successful in Europe with a number of pregnancy cohorts that are currently ongoing. However, some investigated exposures may have become less important, e.g., recently regulated chemicals, while issues tied to contemporary lifestyles or types of work, e.g., the use or abuse of technological resources, may be lacking. Thus, depending on the study focus, further pregnancy cohort studies could still be warranted.

Moreover, we conducted a number of evaluations showing the scientific profiles of pregnancy cohorts in Europe. Specifically, we showed that two different profiles are commonly observed based on key scientific areas. A more common profile is the one in which the topic is related to lifestyle and nutrition while investigations on environment, allergy and neurodevelopment are less common. According to our results, these cohorts collect both objectively assessed and self-reported data, with a preference for objectively assessed approaches. Self-reported data, as opposed to objectively assessed data, can introduce bias, but pregnancy cohorts have the advantage of collecting data directly during pregnancy and are thus able to overcome methodological limitations such as recall bias for exposures and confounding variables [20,21].

The main key areas of interest in the pregnancy cohorts reviewed were lifestyle, environment, and nutrition, and two-thirds of the cohorts covered all three. Almost all cohorts investigated more than one area, although some were seemingly restricted to specific areas, such as the FONIA cohort study [22], which studied the association between allergen-induced IL-10 production by cord blood cells and prospective risk of allergy development. When looking at the main aims specified by the cohorts, eight addressed intrauterine exposure, focusing on environmental pollutants such as endocrine-disrupting chemicals. These compounds have been studied greatly, but research on their effects on the developing foetus is still needed [23].

[REF: Street et al. (https://pubmed.ncbi.nlm.nih.gov/32093249/, accessed on 10 June 2021)] and the prospective, longitudinal study design of pregnancy cohorts is the optimal strategy for implementing it. With the large use of resources employed in running cohorts and the fact that it is increasingly acknowledged that multiple factors from different fields interact in shaping future offspring health, i.e., the DOHaD concept, cohorts need to be multidisciplinary. Pregnancy cohorts, for example, should also collect preconception data from mothers and fathers, such as socio-economic status and lifestyle, although this would entail linking data to existing population data registries, as is common in northern Europe, or depending to a greater extent on the collection of self-reported data from mothers, possibly introducing some additional bias to the cohort data. Along the same lines, cohorts, in general, should address different fields simultaneously. This comprehensive perspective on health and disease also carries with it responsibilities in performing research and implementing the knowledge acquired in society and policy in “socially just and scientifically robust ways” [24]. In this context, international efforts to group data from different cohorts, such as the EU Child Cohort Network (https://lifecycle-project.eu/for-scientists/the-eu-child-cohort-network, accessed on 10 June 2021), are fundamental to enabling pioneering research on the identification of novel markers of early-life stressors related to health throughout the full life cycle.

A large number of the cohorts reviewed aim to investigate genetics through the collection of biological samples such as blood and urine from the mothers and children. This is in line with research’s priorities based on the evolving knowledge that embedded in the concept of mother to child transmission of genetic information, antibodies, microbiota, etc., lies much more than we are currently are aware of [25]. An area that has been abundantly studied, but in which many unexplained issues still remain, for example, is the relationship between maternal vitamin D status and effects on later offspring neurobehavioural and metabolic health [26]. The research in the fields of DOHaD and epigenetics is fundamental for addressing well-known, but also new, unfamiliar challenges [27,28].

Pregnancy and birth cohorts have an undeniably important role in studying diverse areas, from the well-known ones to emerging issues such as the COVID-19 pandemic. The current and future cohorts will unquestionably study the effects on mothers, pregnancy, and children of the numerous initiatives taken worldwide to contrast the pandemic, from the lockdowns to the vaccines. In fact, the capability of cohorts to observe a large array of effects contemporaneously and in a large group of people, along with the possibility to merge data between international cohorts to observe and compare different settings and even larger populations, places them at the forefront in such uncharted situations where the uncertainty of effects is ubiquitous.

### Strengths and Limitations

The present study has numerous strengths. Among others, we would highlight the rigorous approach and the systematic collection of the data. Of significance is the original approach we used to define the profile of the birth cohorts, along with the rigorous multivariate clustering and network analyses to describe the cohorts’ features. We believe that our work is valuable since it also represents a unique attempt to describe the state of the art of pregnancy cohorts in Europe. Moreover, this work may represent a valid input for the design and conduct of new pregnancy cohorts according to the current European benchmark. Despite numerous strengths, the current work is not without limitations. Firstly, we included a large number of pregnancy cohorts such that we were unable to describe the included cohorts in-depth with our available resources. Secondly, we decided against a comparative approach to avoid possible controversy about study quality. However, a comparative evaluation could have been of use for some readers. Finally, our work focuses on Europe only while it would have been of even greater interest to investigate the pregnancy cohorts’ state of the art also in other continents or macro areas. Widening the geographical scope would, nonetheless, inevitably result in a yet larger number of cohorts to describe; an undertaking too massive for a single study.

## 5. Conclusions

Numerous cohorts exist in Europe and are evolving based on expanding knowledge, e.g., the DOHaD concept, with a consequent increase in the collection of genetic material and in the number of areas studied by individual cohorts [19]. This capability of cohorts to study a large array of effects contemporaneously permits them to focus on specific issues when needed, including emerging situations or factors, such as the SARS-CoV-2 virus and the COVID-19 pandemic in general, with its short and long-term consequences. Few international efforts seem to be in place for now. Merging data would permit the comparison of different settings and populations and would lead to even more robust conclusions, so combined efforts should be encouraged and promoted. Cohort studies and paediatric epidemiology represent an important field of research since preserving healthy lives from conception onwards is the most efficient way to improve population health. For this reason, we sincerely wish that our work could be at least of some inspiration to perform a similar evaluation on birth cohort studies conducted in other continents or macro areas.

## Figures and Tables

**Table 1:** Primary aims of the 66 pregnancy cohort studies.

Cohort/Acronym	Primary Aim
ABC	To investigate the role of genetic factors, environmental exposures and lifestyles in pregnancy on the risk of disease in the offspring
ABCD	To investigate children’s health at birth as well as in later life, and ethnic disparities therein
ALADDIN	Elucidate the role and timing of the anthroposophic lifestyle for the development of allergic disease in childhood
ALSPAC	To identify features of the environment, genotypes and the interaction between the two that influence the health, development and well-being of children throughout the life course
BaBi study	To disentangle the effects of individual and contextual factors among the offspring of migrants and non-migrant. To untangle the underlying reasons for health inequalities between different population groups
Baseline	To establish a prospective paediatrics birth cohort that will have access to detailed information on maternal health, fetal growth and childhood nutrition, growth and development in the first 2 years of life
BASIC	To enrich and expand our knowledge on the pathophysiological processes underlying perinatal depression (PPD) and also pinpoint both epidemiological and biological predictors of the disease, to improve early detection
BPC	The study was originally designed to identify early markers for subsequent pregnancy complications
BiB	To examine how genetic, nutritional, environmental, behavioural and social factors impact health and development during childhood, and subsequently adult life in a deprived multi-ethnic population
BILD	To study physiological properties of the respiratory system and environmental and genetic risk factors affecting lung development in healthy individuals as well as in preterm subjects from infancy through childhood in relation to wheeze
BraMat	To investigate the effect of prenatal exposure to PFAS on responses to pediatric vaccines and immune-related health outcomes in children up to 3 years of age
CHEF	Prenatal and postnatal exposure to methylmercury and organochlorines related to neurodevelopment
COMBINE	To examine associations of early nutrition, feeding behaviour and early life events with growth, lean and fat mass accrual as well as neurological development to better understand the factors that promote healthy growth and development
COPSAC-2010	How human micro-biome and maternal nutrition during pregnancy interact with the genetic predisposition to cause an abnormal immune modulation in early life towards a trajectory to chronic inflammatory diseases such as asthma and others
CRIBS	To assess the prevalence of risk factors (biological, environmental and behavioural) for the Metabolic Syndrome (MetS)
DNBC	General, multiple aims to investigate the causal link between exposures in early life and disease later on and the possibilities for disease prevention
DBC	Investigate prenatal exposure to PCDD/Fs and dioxin-like PCBs in association with thyroid hormone status
EDEN	To study the pre and postnatal determinants of the child’s development and health
EHL	To investigate the impact of gestational and postnatal environmental risk factors on infant health and development across the life course
ELSPAC	To determine which biological, psychological, social, economic and environmental factors have an impact on pregnancy, birth, adaptation after birth, child development, health of children and adolescents
FCOU	To study the biological, psychological and social factors, as well as factors of the external environment, which are associated with survival and health of the fetus, infant and child
FinnBrain	To study prospectively the effects of early life stress, also comprising prenatal stress, on child brain development and health
FONIA	Association between allergen-induced IL-10 production by cord blood cells and prospective risk of allergy development
GECKO	To study the prevalence and early risk factors for the development of childhood overweight and fat distribution at a very young age
Generation R	To identify early environmental and genetic causes of normal and abnormal growth, development and health during fetal life, childhood and adulthood
HHf2	To investigate smoking, drinking and eating behaviour in association with child health
INMA	To describe the degree of individual prenatal exposure to environmental pollutants and the internal dose of these chemicals during pregnancy, at birth and during childhood
IOWBC	To investigate the prevalence, natural history and risk and protective factors for the development of asthma and allergic diseases
IVAAQ	To collect information about pregnant women and their newborn children to study the effects of selected exposures on pregnancy outcome and child development
KANC	To assess the distribution of risk factors for adverse pregnancy outcomes in pregnant women with detailed information on demographic and lifestyle characteristics, medical history, and genetic to define susceptibility to long-term environmental exposure
KOALA	Allergy and asthma development
Krakow Cohort	To test the hypothesis that prenatal exposure to airborne polycyclic aromatic hydrocarbons adversely affects fetal growth, after controlling for non-PAH components of particulate matter <2.5 µm (PM2.5), environmental tobacco smoke, nutritional status
KUBICO	To investigate genetics and stress factors during pregnancy in association with the health status of the mother and child
Life Child	To investigate determinants of healthy child development from pregnancy to adulthood
Lifelines NEXT	To investigate the effects of early life or pre-conceptional transgenerational events on health in early childhood
LIFEWAYS	To record the physical and psychological health status and socio-economic circumstances of individuals at birth, during early childhood, early adulthood and early middle age
LINA study	To investigate prenatal lifestyle and environmental factors in association with newborn allergy
LINC	To study associations between early-life environmental exposures and child health, including growth and neurodevelopment
LUKAS	Environmental exposure: microbes from stables and from moisture damaged homes, asthma, sIgEs, atopic eczema, hayfever, respiratory symptoms, development of the immune system
MAAS	To investigate the risk factors for the development of asthma and other atopic disorders
MAMI	To characterize the microbial community, diversity and activity in mother-infant interphase by use of both, culture-dependent microbiological techniques and culture-independent methodologies based on PCR, from stool, oral and breastmilk samples collected during the first two years of infant life
MEFAB	To investigate whether prenatal availability of long-chain polyunsaturated fatty acids (LCPUFAs) may be involved in programming birth outcomes and later development
MAS	To study prospectively the development of allergy in relation to infant feeding and various environmental factors
MoBa	To find causes of diseases
NELA	To investigate the impact of nutrition during pregnancy and early postnatal life on health outcomes in children
NICE	To evaluate how multiple environmental exposures such as lifestyle, diet, microbes and toxicants influence the maturation of the immune system and affect allergy development
NINFEA	To investigate the effects of exposures acting during pre-natal and early post-natal life on infant, child and adult health
NFBC	To investigate factors in association with pre-term birth and intrauterine growth retardation. Investigate the consequences of these early adverse outcomes on subsequent morbidity
OCC	To assess environmental factors in pregnancy and early childhood and their impact on child health
PASTURE	To identify mechanisms of early protective exposures on allergy development using birth cohort studies with comprehensive measures of the developing immune response
PELAGIE	To investigate long-term consequences of prenatal and early childhood exposure to the endocrine-disrupting chemicals
PHIME	To describe the degree of individual prenatal exposure to environmental pollutants and the internal dose of these chemicals during pregnancy, at birth and during childhood
PIAMA	To investigate the effect of mite-allergen avoidance on the incidence of childhood asthma and to assess lifestyle and environmental risk factors for childhood asthma.
Predict Study	To investigate failures and adverse pregnancy outcomes and health of the offspring up to 1 year of age
PRIDE Study	To identify factors to which women may be exposed during pregnancy that potentially affect the health of the future mother or her unborn child at any point in life. To evaluate specific aspects of preconceptional, prenatal and perinatal care
REPRO_PL	To evaluate the impact of exposure to different environmental factors during pregnancy and after birth on pregnancy outcome and children’s health
RHEA	To characterize nutritional, environmental and psychosocial determinants of children’s growth and development, to examine: (i) growth and physical development; (ii) behavioural, cognitive and sociο-emotional development; (iii) allergies
SEATON	To study maternal dietary antioxidant intake and atopic disease during childhood
SESBiC	To assess whether mothers who reported symptoms of PPD were more likely than others to report depressive symptoms 12 years later
SBC	To evaluate and quantify how various modifiable environmental and dietary exposures contribute to the development of infantile atopic eczema
SNiP	To investigate biological features from mothers in association to neonatal health, morbidity and mortality and child health prior to attendance at primary school
STEPS	To contribute to a comprehensive, integrated view of healthy development: to improve the understanding of the early development of children, and their health and well-being beginning from the prenatal period up to school age, and also to determine the long-term effects of their early development later on in life
SWS	Maternal pre-conceptional and pregnancy factors: diet, physical activity, body composition, hormonal levels, genetics, epigenetics. Childhood growth, respiratory health, body composition, cardiovascular health
TI-MOUN	To investigate perinatal exposure to chlordecone in association with thyroid hormone status and neurodevelopment in infants
UBCS	Investigate lifestyle factors of mother in association to child health
WCHDS	To identify the very early risks and processes in the development of behavioural and emotional problems in childhood

Notes: ABC—Aarhus birth cohort; ABCD—The Amsterdam Born Children And Their Development Study; ALADDIN—Assessment of Lifestyle and Allergic Disease During Infancy; ALSPAC—Avon Longitudinal Study of Parents and Children of the 90S; BaBi study—Bielefeld birth cohort; BASELINE—Babies after scope: Evaluating the longitudinal impact using neurological and nutritional endpoints; BASIC; Berlin Pregnancy Cohort; BiB—Born in Bradford; BILD—Bern-Basel-Infant Lung development cohort; BraMat; CHEF-1 Children’s Health and the environment in the Faroes; COMBINE-The Cork Nutrition Maternal-Infant Cohort Study; COPSAC-2010- Copenhagen Prospective Studies on Asthma in Childhood; CRIBS—Croatian Island’s Birth Cohort study; DNBC—Danish National Birth Cohort; DBC—Duisburg Birth Cohort Study; EDEN—The Study Of Pre And Post Natal Determinants Of Child Growth Development And Health; EHL—Growing Up in Wales: environments for healthy living ; ELSPAC—The European Longitudinal Study of Pregnancy and Childhood In the Czech Republic; FCOU-Families and children of Ukraine; FinnBrain Birth Cohort Study; FONIA Study; GECKO—Groningen expert center for kids with obesity Drenthe cohort; Generation R; HHf2-Healthy Habits for two; INMA—The Environment And Childhood Project; IOWBC—population based cohort established 1989 on the Isle of Wight, UK; IVAAQ—The Greenland Child Cohort; KANC—Kaunas Cohort; KOALA Birth Cohort Study; Krakow Cohort; KUBICO—Kuopio birth cohort; Life Child; Lifelines NEXT; LIFEWAYS Lifeway Cross-Generation Cohort Study; LINA study (Lifestyle and Environmental Factors and their Influence on Newborn Allergy risk birth cohort study).; LINC study; LUKAS; MAAS—Manchester asthma and allergy study; MAMI- Maternal-infant microbiota during early life; MEFAB—Maastricht Essential Fatty Acid Birth cohort; Merthyr Allergy Study; MoBa—Norwegian Mother and Child Cohort; NELA—Nutrition in early life and asthma; NICE—Nutritional impact on Immunological maturation during Childhood in relation to the Environment; NINFEA—Nascita e INFanzia, gli Effetti dell’Ambiente; NFBC—Northern Finland Birth Cohort 1966; OCC—Odense Child Cohort; PASTURE project; PELAGIE—Endocrine distruptors:longitudinal study on pathologies of pregnancy, infertility and childhood; PHIME—Public Health Impact of long-term, low-level, Mixed Element exposure; PIAMA—Prevention and incidence of asthma and mite allergy; Predict Study-Rotterdam periconceptional cohort study; PRIDE Study—Pregnancy and infant development study; REPRO_PL -Polish National Mother and Child Cohort; RHEA—Mother and Child Cohort on Crete; SEATON—Study of eczema and asthma to observe the effects of nutrition; SESBiC—study South East Sweden Birth Cohort-study; Slovak birth cohort; SNiP—Survey of Neonates in Pommerania; STEPS—Steps to the healthy development and well-being of children; SWS Southampton Women’s Survey; TI-MOUN; UBCS-Ulm birth cohort study; WirralA12:A68 Child Health and Development study.

## Data Availability

Current data are not available.
